# Practical Aspects of Using Multifractal Formalism to Assess the Morphology of Biological Tissues

**DOI:** 10.17691/stm2023.15.3.04

**Published:** 2023-05-28

**Authors:** A.M. Ignatova, M.A. Zemlyanova, O.B. Naimark, N.V. Zaitseva

**Affiliations:** Senior Researcher; Institute of Continuous Media Mechanics of the Ural Branch of the Russian Academy of Sciences — а Branch of the Perm Federal Research Center, Ural Branch of the Russian Academy of Sciences, 13A Lenina St., Perm, 614990, Russia; Researcher; Federal Scientific Center for Medical and Preventive Health Risk Management Technologies, 82 Monastyrskaya St., Perm, 614045, Russia;; Associate Professor, Chief Researcher; Federal Scientific Center for Medical and Preventive Health Risk Management Technologies, 82 Monastyrskaya St., Perm, 614045, Russia; Professor, Department of Environmental Protection; Perm National Research Polytechnic University, 29 Komsomolsky Prospekt, Perm, 614990, Russia; Professor, Head of the Laboratory; Institute of Continuous Media Mechanics of the Ural Branch of the Russian Academy of Sciences — а Branch of the Perm Federal Research Center, Ural Branch of the Russian Academy of Sciences, 13A Lenina St., Perm, 614990, Russia;; Academician of the Russian Academy of Sciences, Scientific Adviser; Federal Scientific Center for Medical and Preventive Health Risk Management Technologies, 82 Monastyrskaya St., Perm, 614045, Russia;

**Keywords:** image analysis, histology, lung tissue, fractal analysis, alveolar pattern

## Abstract

**The aim of the study** is to identify practical aspects of using multifractal formalism to assess the morphology of biological tissues.

**Materials and Methods:**

The objects of the study were histological images of lung tissues of Wistar rats without pathology and with detected pathological changes, obtained at 50×, 100×, 200× magnifications. Image processing was carried out using the ImageJ/Fiji universal software. The multifractal spectrum of the images, processed to obtain a linear contour, was calculated with the use of FracLac — a module for ImageJ. This module was used to determine the scaling exponent (the function of the Rényi exponent, ^τ^(q)) and the singularity spectrum itself.

**Results:**

The singularity spectra for tissues with no pathology have signs of multifractality. The image spectrum of tissue with pathology is shifted to the left relative to the spectrum characteristic of tissue without pathology. A decrease in the spectral height in the presence of pathology indicates a “simplification” of the alveolar pattern, which is presumably associated with the presence of widespread vasculitis, since it causes areas of hemorrhage to appear on the image; this leads to leveling the contour of the alveolar pattern, reducing the surface area of the alveoli and emerging areas inflamed by erythrocytes. At lower magnification, images with pathology lose signs of multifractality.

**Conclusion:**

Correct results of assessing multifractal spectra of histological images can be achieved at 200× magnification and preprocessing to obtain linear contours. Significant differences between the morphological structure of lung tissues with and without pathology are observed when comparing the height, width, and position of the spectrum relative to the origin.

## Introduction

In terms of classical concepts, histological analysis does not imply obtaining calculable indicators, however, in recent years, in addition to it, image analysis is increasingly used to obtain quantitative characteristics [1-3]. Quantitative characteristics provide the comparison of the results of a large number of experiments and their application for modeling [4-5]. When quantifying the morphology of biological tissues, the problem of determining evaluation criteria arises. Currently, various parameters for the analysis of histological images, in particular, a degree of tissue differentiation based on the Gleason grading system, are used in scientific and clinical practice [6]. This approach and others close to it are applicable only to the assessment of specific pathologies, such as neoplasms, and not suitable for diagnosing and classifying other changes. In a number of works [7-9], in investigation of non-tumor pathologies, the criteria for studying images of biological tissues by means of image analysis are discussed. It is noted that fractal dimension is applicable to assess the organization of neurons, and indicators of dendritic geometry are used to describe the alveolar pattern.

The structure of histological images often cannot be described by a single fractal set. It is a complex of several fractals superimposed on each other, each of which has its own dimension [10]. Such a multifractal nature of histological images is due to the fact that biological processes develop according to power laws and, therefore, their phase portraits are characterized by a non-uniform distribution of a certain measure, which can be described using not one, but several scales [7]. That is why a promising method for obtaining image characteristics is multifractal formalism [11-13].

The characteristics of the multifractal spectrum of biological tissue images are proposed to be used as a universal evaluation approach [8].

Direct calculation of the multifractal spectrum of histological images, as a rule, is ineffective due to the presence of artifacts that can lead to an unreliable result, for this reason the use of the multifractal formalism requires specialized image creation. The search for methodological approaches to the use of multifractal image analysis in relation to the assessment of the morphology of biological tissues and objects is relevant.

**The aim of the study** is to identify practical aspects of using multifractal formalism to assess the morphology of biological tissues.

## Materials and Methods

**Figure 1.**
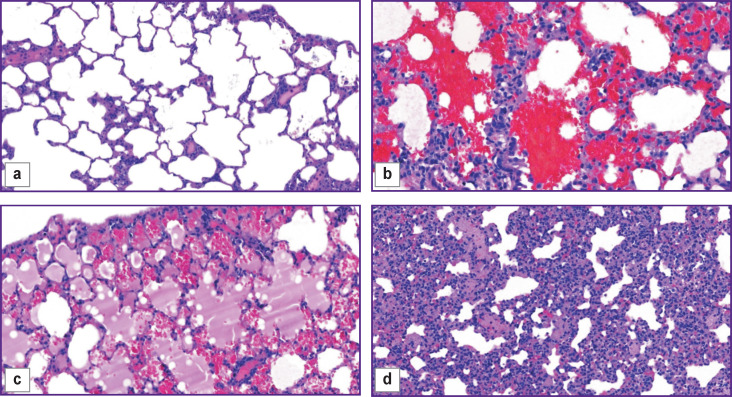
Lung tissue of Wistar rats: (a) without pathology; (b) with hyperplasia of the lymphoid tissue associated with the bronchial wall and widespread vasculitis; (c) with alveolar pulmonary edema; (d) with interstitial pneumonia; ×200

The objects of the study were histological images of lung tissues of Wistar rats without pathology and with detected pathological changes (Figure 1), obtained at 50×, 100×, 200× magnifications. Histological material was obtained from experiments, the conditions of which met the requirements of the European Convention for the Protection of Vertebrate Animals used for Experimental or Other Scientific Purposes (ETS No.123), and the Ethics Committee of the Federal Scientific Center for Medical and Preventive Health Risk Management Technologies (Perm, Russia).

The study examined images of lung tissues with diagnosed hyperplasia of lymphoid tissue associated with the bronchial wall with an admixture of eosinophils and widespread vasculitis, interstitial pneumonia, and alveolar pulmonary edema.

The images were obtained using microscopy of lung tissue specimens. For their making, the organs were fixed in 10% aqueous neutral formalin solution and then dehydrated in ascending alcohols, then soaked in chloroform and paraffin, and poured with Histomix homogenized paraffin medium (BioVitrum, Russia). 4-μm thick histosections were obtained using a JUNG SM2000R sledge microtome (Leica Microsystems, Germany) and stained with hematoxylin and eosin. Inspection and imaging of the morphological structure of tissues was performed using an Axiostar light-optical microscope (Carl Zeiss, Germany).

It is noted in [14] that one of the possible means of minimizing the effect of artifacts in the image analysis is binarization, edge detection and its skeletonization. Image processing was performed using ImageJ/Fiji universal software (open-source software, developed by Wayne Rasband, National Institutes of Health, USA).

**Figure 2.**
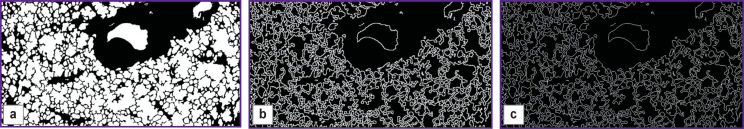
Lung tissue images of Wistar rats prepared for analysis: (a) the result of binarization; (b) the result of edge detection; (c) the result of contour skeletonization

Before making a multifractal analysis, the images were converted to grayscale black and white, and then to two-color ones by the binarization method with a threshold value of 220 (Figure 2 (a)).

The value of lacunarity was estimated for the binary images. This indicator is a measure of the heterogeneity of filling the space with a pattern, which in this case is an alveolar pattern. An increase in the lacunarity value indicates an increase in the number of chaotic dips in the pattern.

In this study, the measure of lacunarity (*L*) is a change in image density when scanning it with a grid with cells of various sizes (square cells ranging in size from 1 to 60 pixels were used). The following formula is used to calculate lacunarity:

L=(σ/μ)2,

where σ is the standard deviation of the number of pixels (for a binary image) of a fractal aggregate in grid cells of a given size; μ is the average value of the mass of the aggregate or the average number of pixels in cells of a given size.

Lacunarity was determined by the slope of the regression line in the coordinates of ε/*L* (ε is the grid cell size in fractions of the raster size) and by the range of *L* values for ε=0.01÷0.6. When graph plotting, the lacunarity was calculated by the formula

L=(σ/μ)2+1,

where σ is used to plot the graph uniformly at zero standard deviation (σ=0).

The edge detection of the resulting binary images was performed using the outline function (Figure 2 (b)), and then its skeletonization was carried out (Figure 2 (c)). As a result of processing, the images of lung tissues presented images of a double linear contour of the alveolar pattern, which were used to obtain a multifractal spectrum. To calculate the multifractal spectrum of images processed to obtain a linear contour, we used the module for ImageJ — FracLac. Using this module, the scaling exponent (the function of the Rényi exponent, τ(q)) and the singularity spectrum were determined. The following parameters were determined for the singularity spectrum: the width and height of the spectrum; the width and height of the right and left branches [15].

## Results and Discussion

**Figure 3.**
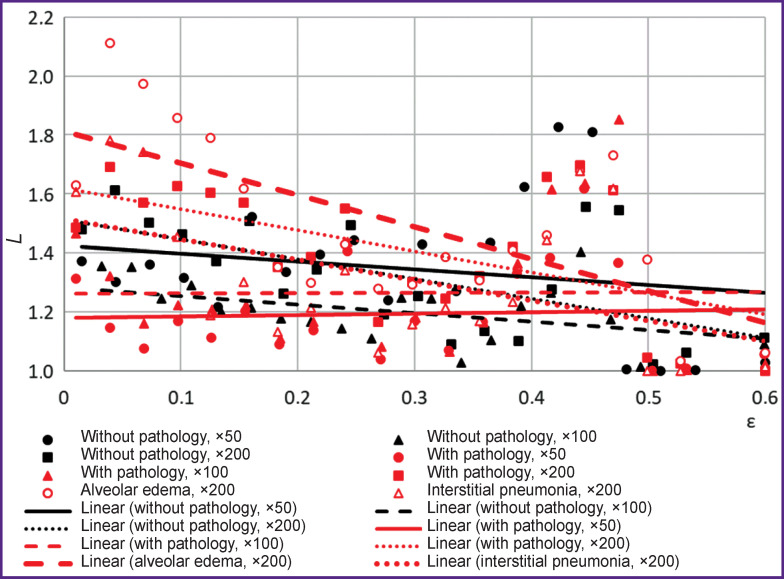
Image lacunarity functions

**Figure 4.**
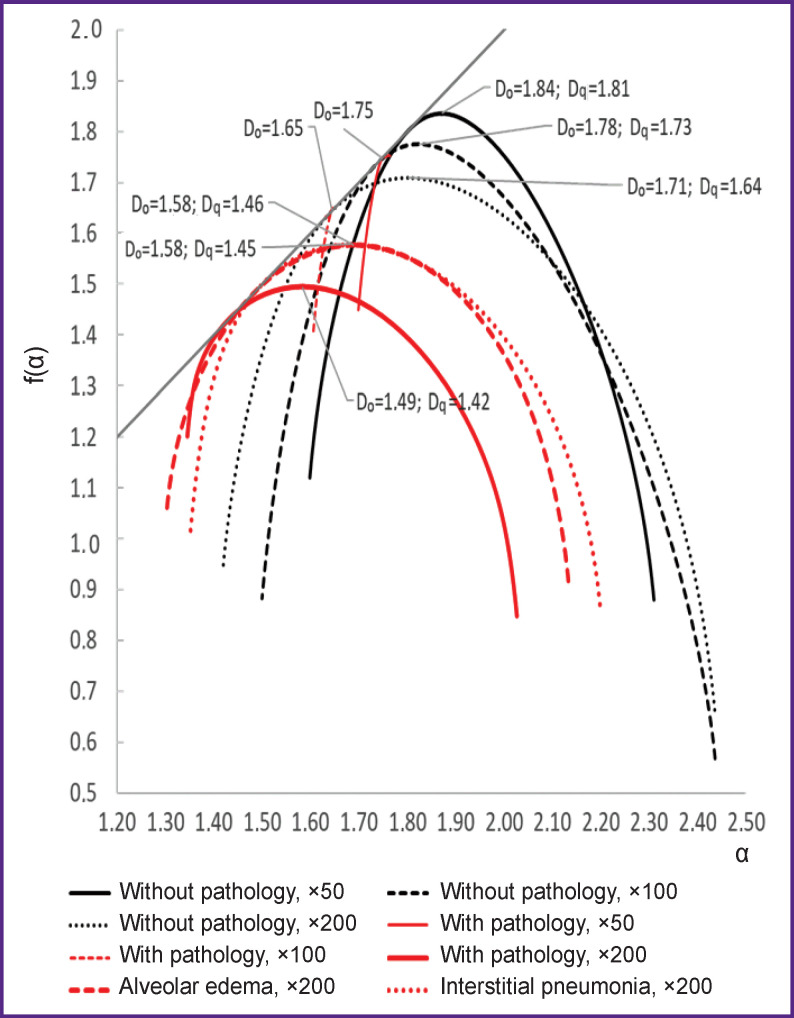
Image singularity spectra D_0_ — monofractal dimension, D_q_ — informational dimension

The results of assessing the lacunarity of the alveolar pattern are shown in Figure 3 and in Table 1. Figure 4 shows singularity spectra characteristic of processed images of histological lung tissues; the spectra characteristics are given in Table 2.

**Table 1:** Lacunarity characteristics of the alveolar pattern

Image characteristics	Lacunarity characteristics		
	Coefficient of the slope of linear regression	Minimum value	Maximum value
***Without pathology***			
×50	–0.26	1.0	1.83
×100	–0.29	1.01	1.40
×200	–0.66	1.02	1.66
***With pathology***			
×50	0.04	1.0	1.61
×100	0.01	1.0	1.85
×200	–0.71	1.0	1.69
Alveolar edema, ×200	–1.08	1.03	2.11
Interstitial pneumonia, ×200	–0.69	1.0	1.78

**Table 2:** Multifractal spectra characteristics of histological images

Image characteristics	Spectrum characteristics					
	Spectrum width, α_max_–α_min_	Spectrum height, f(α_0_)	Width of the right branch, α_max_–α_0_	Height of the right branch, f(α_0_)–f(α_max_)	Width of the left branch, α_0_–α_min_	Height of the left branch, f(α_0_)–f(α_min_)
***Without pathology***						
×50	0.71	1.83	0.44	0.95	0.27	0.71
×100	0.93	1.77	0.61	1.20	1.32	0.89
×200	1.01	1.70	0.63	1.04	0.38	0.77
***With pathology***						
×50	Monofractal pattern					
×100	Monofractal pattern					
×200	0.68	1.49	0.44	0.64	0.24	0.29
Alveolar edema, ×200	0.83	1.57	0.44	0.65	0.38	0.52
Interstitial pneumonia, ×200	0.84	1.57	0.51	0.70	0.33	0.56

**Figure 5.**
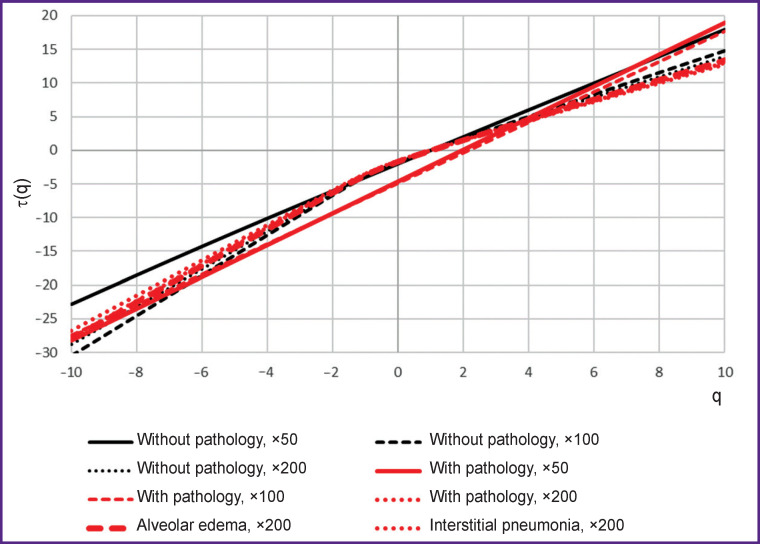
Scaling exponents of images

For tissues with pathology, the lacunarity degree of the alveolar pattern was lower than that for tissues without pathology. A local increase in the lacunarity in the range of ε=0.4÷0.5 is apparently associated with the physiological characteristics of the tissues, namely with the appearance of blood vessels in the alveolar pattern. To the greatest extent, the value of lacunarity identifies alveolar edema. For images of tissues with pathology at 50× and 100× magnification, the lacunarity index is close to zero, which, along with the linear nature of the dependence of the scaling exponents for these images (Figure 5), is a sign of monofractality. At 200× magnification, the singularity spectrum for tissues with pathology, on the contrary, has pronounced signs of multifractality, but the characteristic of the spectrum differs from similar indicators characteristic of tissues without morphological pathologies. In the presence of pathology, the height of the spectrum was lower by 0.2; the spectrum width was more by 0.3; at the same time, the ratio between the widths of the right and left branches remained similar to that observed for tissues without pathologies.

For tissues with no pathology detected, the singularity spectra have signs of multifractality, regardless of the magnification at which the images were obtained. However, with an increase in the degree of magnification of histological images the parameters of the spectra change, namely, the width of the spectrum uniformly increases and its height decreases; at the same time, the ratios of the parameters of the right and left branches are preserved. At 200× magnification, the height of the right and left branches slightly decreases relative to similar figures observed at 100× magnification. These changes in the spectra with increasing magnification correspond to a decrease in the number of alveoli in sight and a more distinct contour of the alveolar pattern.

On the whole, the image spectrum of tissue with pathology is shifted to the left relative to the spectrum characteristic of tissue without pathology. The latter, together with a histological analysis, indicates the growing dominance of lymphoid tissue, which is characteristic of its pathological hyperplasia.

A decrease in the width of the singularity spectrum, which is characteristic of images with pathology, indicates a more uniform distribution of the structural components of such a tissue than tissues without pathology, i.e. the periodicity (rhythm) of the pattern of the alveolar pattern is disturbed; in relation to the lung tissue, this indicates a reduction in the alveolar lumens. A decrease in the height of the spectrum in the presence of pathology indicates a “simplification” of the alveolar pattern, which is presumably associated with the presence of widespread vasculitis, since it causes areas of hemorrhage to come in sight on the image. This leads to the alignment of the contour of the alveolar pattern, the surface area of the alveoli is reduced, and there appear areas filled with red blood cells.

Most notably, at lower magnification, images with pathology lose signs of multifractality. Presumably, the absence of signs of multifractality is also associated with smoothing the contours of the pattern due to the general swelling and focal distribution of erythrocytes. In this regard, the characteristic details are revealed only at higher magnification.

The singularity spectra of images of specified pathological manifestations, namely alveolar edema and interstitial pneumonia, at 200× magnification are close to the spectrum characteristic of pathological changes in general. They have the same height and almost equal width, which, however, is greater than for pathological changes in general, which is associated with a violation of the alternation of alveolar spaces in the pattern characteristic of lung tissues. The spectrum observed in alveolar edema is most shifted to the left, which confirms the assumption of the dominance of a pathological component in the tissue structure.

## Conclusion

Methodological approaches are proposed for using multifractal formalism to assess the morphology of biological tissues. It has been established that the correct results of evaluating the multifractal spectra of histological images can be achieved at 200× magnification and preprocessing to obtain linear contours. Evaluation criteria for the quantitative characteristic of biological tissues by the method of multifractal analysis are the statistical characteristics of the singularity spectrum. Significant differences between the morphological structure of lung tissues with and without pathology are recorded when comparing the height, width and position of the spectrum relative to the origin. Lacunarity is not a universal evaluation criterion.
